# Hydrological controls on river network connectivity

**DOI:** 10.1098/rsos.181428

**Published:** 2019-02-13

**Authors:** Silvia Garbin, Elisa Alessi Celegon, Pietro Fanton, Gianluca Botter

**Affiliations:** 1 Dipartimento ICEA, Università degli Studi di Padova, Padova, Italy; 2 i4 Consulting S.r.l., Padova, Italy

**Keywords:** streamflow dynamics, hydrological connectivity, river fragmentation, ecohydrology

## Abstract

This study proposes a probabilistic approach for the quantitative assessment of reach- and network-scale hydrological connectivity as dictated by river flow space–time variability. Spatial dynamics of daily streamflows are estimated based on climatic and morphological features of the contributing catchment, integrating a physically based approach that accounts for the stochasticity of rainfall with a water balance framework and a geomorphic recession flow analysis. Ecologically meaningful minimum stage thresholds are used to evaluate the connectivity of individual stream reaches, and other relevant network-scale connectivity metrics. The framework allows a quantitative description of the main hydrological causes and the ecological consequences of water depth dynamics experienced by river networks. The analysis shows that the spatial variability of local-scale hydrological connectivity is strongly affected by the spatial and temporal distribution of climatic variables. Depending on the underlying climatic settings and the critical stage threshold, loss of connectivity can be observed in the headwaters or along the main channel, thereby originating a fragmented river network. The proposed approach provides important clues for understanding the effect of climate on the ecological function of river corridors.

## 1. Introduction

River networks are key elements of the landscape, as they represent ecological corridors for biological species and contribute significantly to shape the hydrological response of catchments [1–3]. In the large majority of existing theoretical and experimental works concerning the propagation of waterborne diseases, ecological dispersion and catchment-scale biogeochemistry (e.g. [4–8]), river networks are thought of as static connections between fixed nodes defined on the basis of the topography of the terrain [9–12]. However, empirical observations suggest a dynamic behaviour of the flowing network, which is a reflection of the underlying space-and-time variability of hydrological processes. The continuous expansion and contraction of stream width and depth in response to time-variant hydroclimatic forcing (e.g. rainfall) may create physical disconnections between river segments [13,14]. Therefore, the ecological function of river networks can be significantly reduced by unfavourable local hydraulic conditions that challenge the migration of fishes, propagules and invertebrates, with notable implications for the composition of metacommunities [15–18].

Climate, vegetation and landscape properties determine the natural flow regime of rivers, which their ecological integrity depends upon [19–21]. In view of the role of rivers as ecological corridors, conceptual models of species distribution have been developed addressing spatial and temporal biodiversity patterns in riverine systems [5,22]. However, these studies are based on static networks defined solely on the basis of geomorphological features and, therefore, they cannot capture the effect of hydrological processes on the ecological function of rivers. The intertwined link between reach-scale ecological processes and discharge variability in rivers has received much attention in the literature [23–26]. For instance, several methods based on the physical simulation of habitats have been developed to predict flow-based alteration of habitat characteristics [27–29]. Nevertheless, the study of the network-scale ecological implications of discharge dynamics is a relatively new discipline [14,30]. Most existing studies are focused on individual river reaches and thereby neglect the spatial dimension of rivers.

According to the river continuum concept, rivers are hydrological continua where ecological processes and species dynamics take place [31]. Following this pioneering concept and its successors [32,33], the riverscape paradigm offers a new perspective for integrating ecological processes with spatial dynamics of hydrological regimes [34]. There is growing recognition that river flow regimes control the magnitude of in-stream processes [35], as well as the connectivity between source areas and the catchment outlet, with important implications for biodiversity and ecological functions of rivers across scales [30,36]. For instance, empirical relationships between fluvial species activities and flow variability have been recognized, in particular, as influencing fish and aquatic invertebrate migration [15,25,37,38]. Meanwhile, theoretical approaches have been developed to quantify how the connectivity structure of habitat networks constrains or promotes the ecological function of rivers [39–41]. However, in all these studies a causal connection between river network connectivity and first-order climatic and hydrological drivers is missing, and little is known about the role of hydrological drivers that shape the ecological function of stream networks.

To fill this gap in our knowledge, we propose here a probabilistic framework able to investigate quantitatively the principle of causality that drives the link among the following cascade of processes: (i) climatic driving forces (rainfall and evapotranspiration), (ii) the hydrological response of rivers, (iii) the connectivity of the network structure, and (iv) the fate of ecological species therein. This work is a physically based analytical characterization of streamflow regimes at network scale that explicitly accounts for the randomness of rainfall. The flow regime is defined through the probability distribution of discharge, expressed as a function of lumped parameters that embody long-term climatic and landscape features of the contributing areas. Probability distributions of water stages are consequently derived and used to predict the hydrological and ecological impacts of hydro-climatic fluctuations by means of suitable stage and connectivity thresholds.

## 2. Methods

### 2.1. Streamflow model

The seasonal probability distribution of streamflows is derived by using a mechanistic analytical model which is based on a stochastic description of catchment-scale water storage dynamics. Model details are provided in the following sections. The methods are based on the work proposed by Botter *et al*. [42] and further developed in [43,44].

#### 2.1.1. Rainfall model

The catchment-scale water storage is controlled by the stochasticity of rainfall. In this paper, we have extended a lumped formulation widely used in the literature [45–52] by implementing a spatio-temporal Poisson process for the stochastic generation of daily rainfall. The occurrence of rain events is described by a counting process {N(t,X), t≥0} of rate $\lambda_{\mathrm{rain}}(t,X)>0$, which is a multi-dimensional Poisson process representing the number of rain events occurring per unit time and per unit area. The process is decoupled into two independent Poisson processes, one in time (with rate λ_*t*_) and one in a two-dimensional space (with rate λ_*x*_ λ_*y*_). In order to define the position of the rain events within the domain, the coordinates of the rain cells are assumed to be uniformly distributed across the domain. The model reproduces a precipitation process in which rain events are made up by circular cells of constant duration (1 day) during which random rain depths occur. The radius of each cell is assumed to be an exponentially distributed random variable independent of the other attributes of the cell (e.g. position, intensity), and distributed with the following probability density function (PDF):

2.1 $$p_{r}(r)=\kappa \;\exp^{-\kappa \;r},$$

where the parameter *κ* = 1/〈*r*〉 is the inverse of the mean radius. Likewise, rain intensity within each cell is an exponentially distributed random variable, whose PDF is:

2.2 $$p_{\zeta }(\zeta )=\mu \;\exp^{-\mu \;\zeta },$$

where the rate μ=1/⟨ζ(X)⟩ is the inverse of the mean depth pertaining to each cell centred in X=(x,y). Cells can overlap and the rainfall depth is the sum of the intensity of all cells active at the given time.

Using the rainfall generation model described above, spatially distributed rainfall scenarios are produced to simulate different types of climate (i.e. dry, intermediate and wet [53]), thereby originating different patterns of spatially averaged rainfall depths (*α*[*L*]) and average rainfall frequency (λ_*P*_[*T*^−1^]) along the river network. These are calculated by analysing the time series of synthetic rainfall, spatially averaged over the contributing areas of each network node.

#### 2.1.2. Water balance model

The dynamics of specific streamflow at each node of the network is impacted by positive increments corresponding to rainfall events filling the soil water deficit caused by plant transpiration in the contributing catchment and producing drainage. When the rainfall infiltrating into the hydrologically active layer (i.e. the layer of soil that actively contributes to the hydrological response, whose porosity and depth are indicated as *n* and *Z*_*r*_, respectively) exceeds the critical saturation value *s*_1_ (representing the water-holding capacity), the excess of water becomes streamflows. Note that in between rainfall events the evapotranspiration, ET [*LT*^−1^], reduces the soil moisture to the wilting point *s*_*w*_ (for which ET = 0); hence, the maximum soil water storage capacity available to plants is *w*_0_ = (*s*_1_ − *s*_*w*_)*nZ*_*r*_. Flow-producing rainfall events result from the buffering effect operated by catchments during wetting–drying cycles and they are approximated by a new marked Poisson process, whose frequency is λ < λ_*P*_[*T*^−1^]. The ratio *ϕ* = λ/λ_*P*_ identifies the runoff coefficient (mean discharge scaled to the mean precipitation), which defines the partition of the incoming rainfall into streamflows and ET. *ϕ* is influenced by climate, soil and vegetation features according to the following equation [42,51,54]:

2.3 $$\phi =\frac{D_{I}\gamma^{\gamma /D_{I}}\;e^{-\gamma }}{\gamma \Gamma (\gamma /D_{I},\gamma )},$$

where Γ(·,·) is the lower incomplete Gamma function, *D*_*I*_ is Budyko’s dryness index (the ratio between the mean potential evapotranspiration 〈*PET*〉 and the mean rainfall 〈*P*〉) and *γ* is the maximum soil water storage capacity *w*_0_, normalized to the mean rainfall depth in the contributing catchment, *α*.

#### 2.1.3. Recession flow model

Excess rainfall (fraction of water storage exceeding *s*_1_) is released from the soil as river streamflow following a nonlinear catchment-scale storage discharge relation (i.e. *Q* ∝ *V* ^*β*^) [43,55]. The resulting dynamic of daily specific discharge (i.e. per unit catchment area) at a station is governed by the following equation:

2.4 $$\frac{\text{d}q(t)}{\text{d}t}=-Kq{\left(t\right)}^{a}+\xi_{t},$$

where *K* and *a* are the recession coefficient and the recession exponent, respectively, and *ξ*_*t*_ formally embeds the stochastic increments of *q* induced by effective rainfall pulses. A geomorphological recession flow model is then used to estimate the parameter describing the recession flow behaviour (*a* > 0) resulting from the drainage of the contributing catchment [2]. The procedure is grounded on the idea that the hydrological response is linked to the morphological properties of the hillslope-network system. In this model, the recession rate is directly proportional to the distance of the furthest source from the outlet and the recession flow is controlled by the shrinking of the active drainage network (for further details see [2,51]). As a consequence, the parameter *a* can be estimated from morphological data.

The recession coefficient *K*, which depends on both the network morphology and the moisture of the catchment, is calculated as *K* = *θ*(*α*λ)^1−*a*^ [51], where (*α*λ) is the mean specific discharge, *a* is the geomorphic recession exponent and *θ* is the shrinking rate of the network in between rain events.

#### 2.1.4. Probabilistic description of streamflows

The streamflow PDF at network scale emerges directly as a result of aggregation of spatial heterogeneity of climatic and geomorphic features in the contributing areas of each channel site. This is captured by the proposed model by calculating the parameters expressing the frequency and intensity of effective rain events (λ and *α*) and the recession behaviour (*K* and *a*) for every point along the network, as spatially integrated quantities in the corresponding contributing catchment. There are three different types of PDFs of streamflow, depending on the value of the exponent *a* which determines the rate of decrease of *q* during recessions. The case *a* = 1 implies a linear storage–discharge dynamic (d*q*/d*t* = −*kq* + *ξ*_*t*_) in which the decay of flow between subsequent events is exponential-like. The corresponding steady-state PDF of specific river discharge developed by Botter *et al*. [42] is shown here in non-dimensional form,

2.5 $$p_{Q}(q)=\frac{\Gamma {\left(\lambda /k\right)}^{-1}}{\alpha k}{\left(\frac{q}{\alpha k}\right)}^{\lambda /k-1}\exp\left(-\frac{q}{\alpha \;k}\right).$$

Equation (2.5) represents a Gamma distribution with shape parameter λ/*k* and rate parameter *αk*. The general solution of the PDF for the case *a* ≠ 1, 2 is

2.6 $$p_{Q}(q)=C^{*}q^{-a}\exp\left[-\frac{q^{2-a}}{\alpha K(2-a)}+\frac{\lambda q^{1-a}}{K(1-a)}\right],$$

where $C^{*}$ is the normalization constant, such that $\int_{o}^{\infty }p_{Q}(q)\;\text{d}q=1$. Note that, when *a* < 1, the recession between two subsequent runoff events is faster than that of an exponential function. In this case, there is an atom of probability in *q* = 0 ($p_{o}=C^{*}(K/\lambda )\delta (q)$) that must be added to the continuous part of equation (2.6) as the system tends to remain in a zero-discharge state for some time before experiencing a new jump [43]. Moreover, in the case 1 < *a* < 2, the decay is slower than an exponential function and the system cannot reach the condition of zero discharge, even when the inter-arrival between two rainfall events becomes extremely large. The extension of equation (2.6) to the case *a* = 2 is an inverse Gamma [56], in which the streamflow distribution has a power-law tail much heavier than the exponential tail corresponding to the case *a* = 1.

All these equations were applied pointwise along all the streams of the test catchment. Therefore, model parameters are the expression of climatic and landscape attributes in the contributing catchment and vary in space along the network.

In the framework used in this paper, flow regimes can be classified based on the variability of river flows [21], which results from the interplay between the frequency of flow-producing rainfall events and the mean catchment response time. When the mean inter-arrival of effective rainfall events is shorter than the duration of the flow pulses delivered from the contributing catchment, a persistent supply of water is guaranteed to the stream from catchment soils. This type of regime is termed persistent as the coefficient of variation of streamflow (*CV*_*Q*_) is smaller than 1. On the contrary, when the mean inter-arrival between flow-producing rainfall events is larger than the typical duration of the resulting flow pulses, significant streamflow fluctuations are observed. In this case, the preferential state of the system is typically lower than the mean and the flow regime (termed erratic) is characterized by a pronounced flow variability (*CV*_*Q*_ > 1).

### 2.2. Stage dynamics and connectivity measures

**Figure 1.**
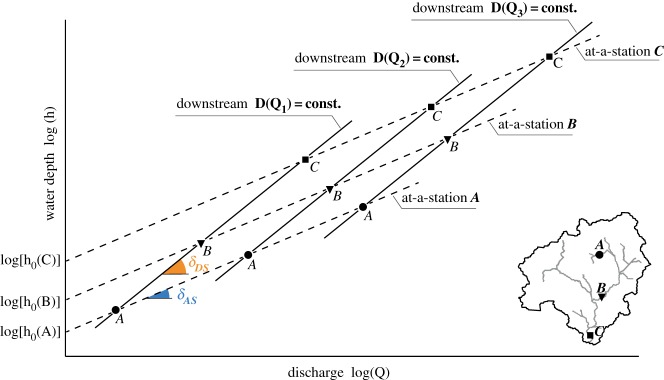
Relation of depth to discharge, for selected cross sections (dashed lines) and in the downstream direction for given discharge frequencies (solid lines).

The temporal and spatial variability of streamflows affects patterns of hydraulic variables (e.g. water depth, flow velocity and bottom shear stress), which influence the distribution of communities and species abundance in fluvial ecosystems [25]. In this work river width, depth and velocity are assumed to increase downstream according to the power-law relationship proposed by Leopold [57]. Accordingly, site-specific PDFs of relevant flow variables can be derived from the corresponding streamflow distribution, *p*_*Q*_(*q*), using additional information on the geomorphic and hydraulic properties of the river cross section. In doing that, the following working hypothesis is introduced: (i) flow conditions are locally uniform, (ii) the river cross section approximates a rectangular shape, and (iii) the water depth is much smaller than the river width. In this framework, water depth (i.e. stage) is assumed to scale with discharge as [57]:

2.7 $$h=h_{0}Q^{\delta }=h_{0}{\left(Aq\right)}^{\delta },$$

where *A* is the catchment area, *h*_0_ is the stage associated with the unitary discharge and *δ* is a dimensionless parameter experimentally found to be close to 0.3 for many rivers worldwide [25,58]. Equation (2.7) represents the ‘at-a-station’ stage–discharge relationship. The parameter *h*_0_, which in general depends on the geometrical characteristic of the cross section, is assumed to be constant and equal to 0.4 [*s*^*δ*^*m*^1−3*δ*^] in this study. The rationale behind this assumption is given below. For a given basin (figure 1), the at-a-station stage–discharge relationship would require that the parameter *h*_0_ scales downstream (i.e. $h=h_{0}(i)Q^{\delta_{AS}}$, where *i* is the considered node); the corresponding downstream relationship (*sensu* [57]), on the other hand, assumes that *h*_0_ is uniform downstream for a given frequency of discharge (i.e. $h=h_{0}Q^{\delta_{DS}}$). Since in most cases the slope *δ*_*AS*_ ≃ *δ*_*DS*_, as demonstrated by Leopold [57], it follows that *h*_0_ in equation (2.7) should be roughly uniform (figure 1) along the network.

By coupling equations (2.6) and (2.7), the following analytical expression of the stage PDF is obtained:

2.8 $$\begin{array}{rl} \;p_{H}(h) & \propto \frac{{\left(h/h_{o}\right)}^{\left(1-a\right)/\delta }}{\delta hA^{\left(1-a\right)}}\exp\left[-\frac{1}{\alpha K(2-a)}{\left(\frac{{\left(h/h_{o}\right)}^{1/\delta }}{A}\right)}^{\left(2-a\right)}+\frac{\lambda }{K(1-a)}{\left(\frac{{\left(h/h_{o}\right)}^{1/\delta }}{A}\right)}^{\left(1-a\right)}\right]. \end{array}$$

Water stage is a major control on the physical connection between two nodes of a river network. For instance, large fish migrating towards headwater streams during drought periods may find it difficult to reach their target in the case where minimum flow requirements are not guaranteed [17,59]. Likewise, many ecological species could be particularly vulnerable to predation during migration in shallow water [60]. Hence, low stages (associated with low flows) can be seen as a physical barrier that decreases the chances of completing migratory movements, with implications for the composition of structured metacommunities [16]. In line with [17], we assume here the existence of a minimum threshold stage, $h^{*}$, which is necessary to trigger the movement of biological species. When $h<h^{*}$, the corresponding stream is assumed to be ‘too dry’ to maintain the connection between upstream and downstream sites. In general, $h^{*}$ is a function of the specific species considered and its sensitivity to droughts. For instance, large fishes are likely to be characterized by larger values of $h^{*}$ than bacteria and propagules.

Considering the connectivity as a categorical and instantaneous variable (connected versus disconnected) is less informative than focusing on temporally integrated quantities such as the frequency and duration of hydrological conditions that allow for species migration ($h\geq h^{*}$). Therefore, we evaluate the connectivity of a given reach by considering the fraction of days within a season during which hydrological conditions favourable to species movement are observed. The latter is calculated as the exceedance probability of the stage threshold $h^{*}$. This probability represents the probability of experiencing water stages that ensure a physical connection between different sites of the network.

Connectivity metrics are based on the mathematical structure of the graph theory. In particular, stream heads and confluences are represented by nodes, whereas branches of the river are seen as links. The relational schema of the graph is undirected, assuming that ecological communities in riverine landscapes can move either upstream or downstream. In this paper, different connectivity measures are used, as follows.

— Local connectivity:
  2.9 $$C_{{\mathrm{local}}_{\left(n\right)}}=\int_{h^{*}}^{+\infty }p_{H}{\left(h\right)}_{\left(n\right)}\;\text{d}h,$$
  where *p*_*H*_(*h*)_(*n*)_ is the stage density function for the *n*-th node. *C*_local_ measures the passage probability through the node (*n*).
— Path connectivity:
  2.10 $$C_{{\mathrm{path}}_{\left(j,k\right)}}=\prod_{n\in j\to k}C_{{\mathrm{local}}_{\left(n\right)}},$$
  where *n* includes the set of nodes belonging to the path that connects node *j* to *k*. *C*_path_ is calculated as the product of the *C*_local_ of all nodes from *j* to *k* and expresses the connection probability between a pair of nodes, according to the hydrological dynamics in all the reaches of the path connecting *j* and *k*. In the calculation of *C*_path_, temporal correlation of flows are neglected.
— Node connectivity:
  2.11 Cnode(k)=1N−1∑ j=1k≠jNCpath(j,k),
  where *N* is the total number of nodes and *j* and *k* are generic nodes of the network. *C*_node_ is calculated as the average value of the connectivity of the paths directed to the node *k*. Accordingly, it expresses the probability for a single node to be connected with all the other nodes of the network.
— Network connectivity:
  2.12 $$C_{\mathrm{ntw}}=\frac{1}{N}\sum_{n=1}^{N}C_{{\mathrm{node}}_{\left(n\right)}}.$$
  *C*_ntw_ expresses the connection probability of all the possible pairs of nodes within the entire river network; *C*_ntw_ is the average value of the probability of connecting any site to all other sites in the network.

### 2.3. Habitat suitability

The spatial variability of streamflow regimes not only affects river connectivity (equations (2.9)–(2.12)), but also influences habitat distributions in rivers [14,25,61,62]. The ecological function of rivers relies on the presence of a mosaic of different habitats connected through the river network. However, the same ecological habitat can be used for different ecological functions (or not), depending on the local streamflow availability. In this work, we include an empirical description of the ecological relevance of each node of the network by considering a local habitat suitability function that accounts for how the ecological functionality of a given site varies in time with streamflow. Habitat suitability curves are a simple tool that describes species habitat preferences under different flow conditions, summarizing the effect of environmental variables on species distribution in rivers [27,63]. In this paper, an empirical Gamma function is used to model the relation between fish habitats and flow availability [64],

2.13 $$\mathrm{HS}(q)=C\exp(-Bq)q^{A-1},$$

where *A*, *B* and *C* are empirical parameters dependent on the channel morphology, water temperature and species length. The average value of the habitat suitability 〈HS〉 is then obtained as

2.14 $$\langle \mathrm{HS}\rangle =\int_{0}^{\infty }\mathrm{HS}(q)p_{Q}(q)\;\text{d}q.$$

Equation (2.14) quantifies the average ability of a given site to provide usable habitats under time-variant flow conditions, taking into account the local flow regime. Therefore, climatic and landscape variables affect, through *p*_*Q*_(*q*), both the connectivity along the network (equations (2.9)–(2.12)) and the average habitat suitability of each node (equation (2.14)).

### 2.4. Estimation of model parameters and simulations set-up

The rainfall model and spatially distributed potential evapotranspiration maps are used to simulate different climate scenarios (appendix A). Climatic fields are then used as an input for the flow model, which is applied to a representative stream network. Model parameters are estimated (for every node of the network) based on the simulated rainfall and other hydrological and geomorphological properties of the upstream contributing catchment. Equation (2.7) is used to derive the spatial and temporal variability of water stages along the network. Then, the hydrological connectivity is calculated using stage thresholds referred to different ecological species. Eventually, the ecological relevance of every node is considered by accounting for the spatial variability of habitats driven by local flow conditions (see appendix A).

## 3. Results

### 3.1. Effects of precipitation and evapotranspiration on network connectivity

Rainfall frequency, intensity and amount are major drivers of the availability and variability of streamflows, and thus they are likely to impact significantly on the hydrological connectivity of rivers. In this section, the network connectivity is studied considering three different rainfall frequencies (e.g. λ_*P*_ = 0.1 d^−1^, λ_*P*_ = 0.5 d^−1^ and λ_*P*_ = 1 d^−1^) under various climatic scenarios in terms of mean precipitation 〈*P*〉 and mean potential evapotranspiration 〈PET〉.

**Figure 2.**
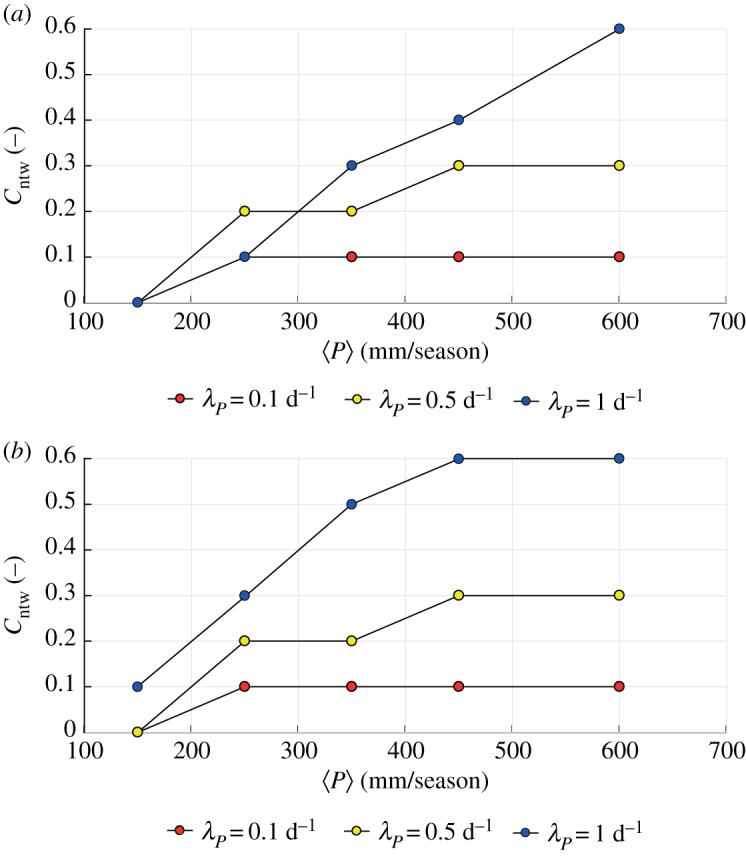
Network connectivity for increasing precipitation and different rainfall frequencies assuming (*a*) 〈PET〉 = 3.5 mm d^−1^ and (*b*) 〈PET〉 = 0.5 mm d^−1^.

The network connectivity *C*_ntw_ typically increases by increasing the mean precipitation depth, if the frequency of the events is constant (figure 2*a*). Moreover, in wet climates (〈*P*〉 > 300 mm/season) connectivity also increases with increasing λ_*P*_ when the precipitation amount is kept constant. For high frequencies of rain events the soil moisture is often close to the field capacity, thereby originating persistent flow regimes (*CV*_*Q*_ < 1) with relatively high flows. Conversely, in intermediate climates (〈*P*〉 = 200−300 mm/season) *C*_ntw_ can increase also when the rainfall frequency is reduced, as low-frequency events have higher intensity (as the rainfall amount is constant). This circumstance reduces the buffering capacity of the catchment and increases the mean streamflow and the mean stage along the network. For low values of 〈PET〉 (0.5 mm d^−1^; figure 2*b*) the connectivity generally increases when: (i) λ_*P*_ is kept constant and 〈*P*〉 increases and (ii) 〈*P*〉 is kept constant and λ_*P*_ increases. The latter mechanism is particularly evident under wet climatic conditions, during which large rainfall inputs inhibit the buffering capacity of the soil, leading to higher mean streamflows and higher connectivities throughout the network.

**Figure 3.**
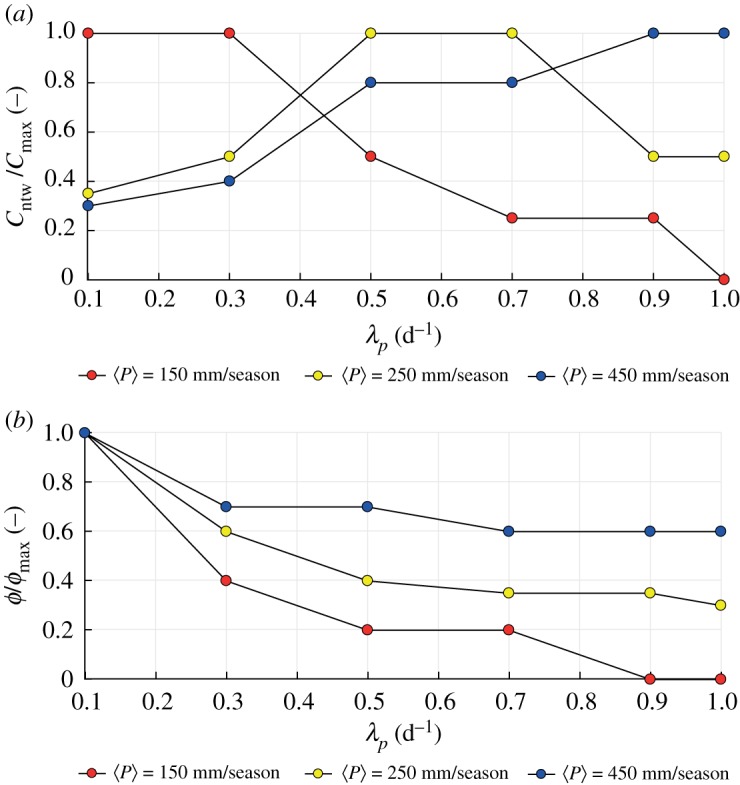
(*a*) The ratio of the network connectivity to the maximum network connectivity for increasing rainfall frequency with different mean precipitation assuming uniform 〈PET〉 = 3.5 mm d^−1^ and (*b*) the ratio of the runoff coefficient to the maximum runoff coefficient for increasing rainfall frequency with different mean precipitation assuming uniform 〈ET〉 = 3.5 mm d^−1^.

In order to evaluate the impact of evapotranspiration on the hydrological connectivity, *C*_ntw_ was calculated assuming different combinations of mean seasonal precipitation and evapotranspiration rate. Figure 3*a* shows how the network connectivity changes as a function of rainfall frequency and for increasing values of 〈*P*〉, combined with a relatively high and uniform 〈PET〉 (3.5 mm d^−1^). In particular, in the wet scenario (〈*P*〉 = 450 mm/season) the connectivity increases with the frequency of rainfall due to the higher mean streamflows associated with larger λ_*P*_. This prevents significant soil water deficits in between events, as confirmed by high values of the runoff coefficient in this case (blue dots in figure 3*b*), and leads to persistent hump-shaped flow regimes, especially in the downstream reaches of the network. Conversely, when the rainfall amount is low (〈*P*〉 = 150 mm/season), the runoff coefficient decreases as the rainfall frequency increases. This suggests that in dry scenarios streamflow regimes could be erratic throughout the river network, with enhanced network fragmentation for larger rainfall frequencies. When 〈PET〉 is reduced to 0.5 mm d^−1^ the connectivity systematically increases for higher rainfall frequencies, regardless of the underlying precipitation amount. In these circumstances, the runoff coefficient *ϕ* slowly decreases with λ_*P*_, though maintaining relatively high values under all climatic scenarios. This typically generates persistent flow regimes, in which the variability of flows decreases as λ_*P*_ increases. The non-exceedance probability of the critical stage $h^{*}$, $P[h<h^{*}]$, is thus reduced and the network connectivity increases.

Further analysis is carried out by evaluating the effect of spatial patterns of evapotranspiration (namely north–south, south–north, east–west and west–east directions) on *C*_ntw_ for each climate scenario. *C*_ntw_ shows similar values regardless of the dominant direction of evapotranspiration gradients. This means that spatial patterns of PET do not affect the average connectivity at the network scale.

### 3.2. Spatial variability of hydrological variables and connectivity along the river network

The analysis of the spatial patterns of hydrological and ecological variables is here performed focusing on a relatively dry climatic setting (i.e. 〈*P*〉 = 150 mm/season and spatially uniform 〈PET〉 = 3.5 mm d^−1^). Three different values of the rainfall frequency are investigated (e.g. λ_*P*_ = 0.1 d^−1^, λ_*P*_ = 0.5 d^−1^ and λ_*P*_ = 1 d^−1^). It is worth nothing that the depth of rainfall is decreasing for increasing the frequency, λ_*P*_, as the mean precipitation (the product of the rainfall depth and the rainfall frequency [65]) remains constant during the simulation.

**Figure 4.**
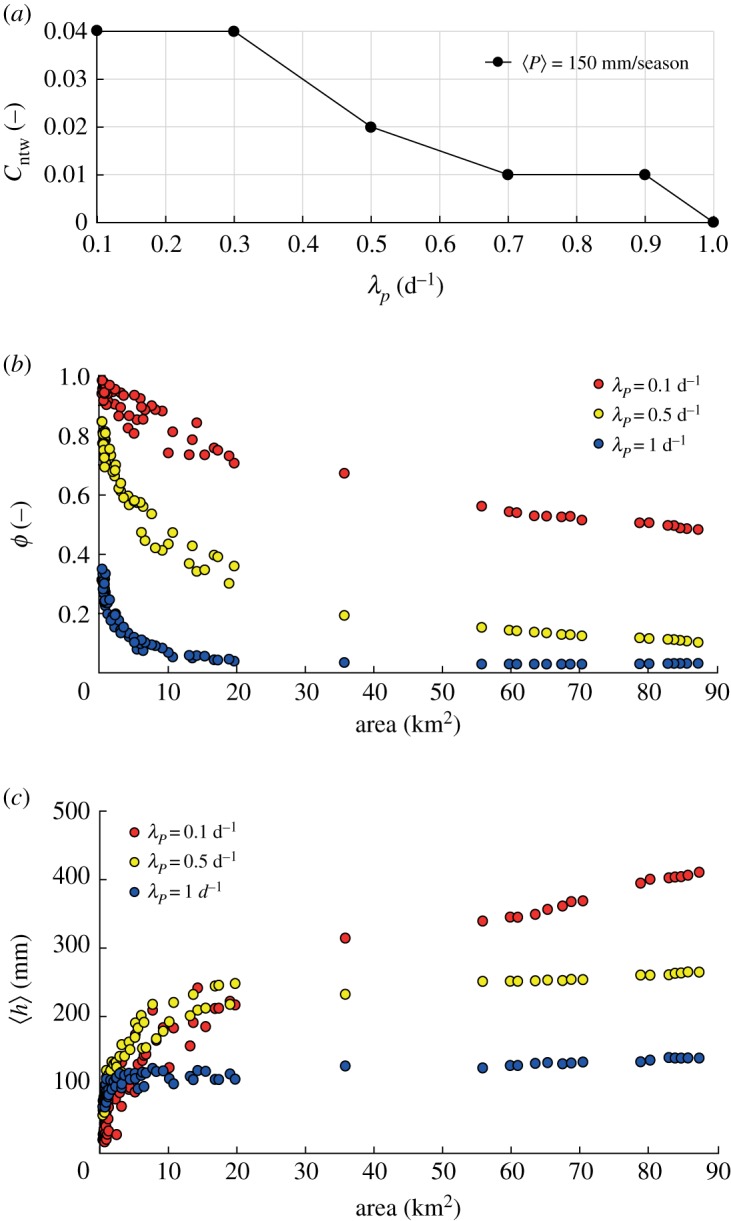
(*a*) Pattern of network connectivity for increasing rainfall frequency; (*b*) scaling relation of the runoff coefficient *ϕ* and (*c*) scaling relation of the mean stage 〈*h*〉. All simulations refer to a dry climatic setting (〈*P*〉 = 150 mm/season and uniform 〈PET〉 = 3.5 mm d^−1^).

Our simulations indicate that the runoff coefficient, *ϕ*, plays a critical role in shaping spatial patterns of connectivity. Under dry climates, *ϕ* generally exhibits a power-law dependence on the drainage area (i.e. *ϕ* ∝ *A*^−*β*^). This is due to the reduction in the mean rainfall intensity and the increase in precipitation frequency for larger contributing areas (the larger the catchment area, the higher the occurrence probability of local rain events that involve only a small portion of the basin). Low rain frequency (λ_*P*_ = 0.1 d^−1^) leads to high values of *ϕ* in downstream sites (figure 4*b*). Hence, the intensity of the events is sufficient to generate persistent flow regimes in most channel sites, thereby increasing *C*_ntw_. Conversely, frequent events with reduced intensity (λ_*P*_ = 1 d^−1^) entail rather small and uniform values of *ϕ* along the network, reducing *C*_ntw_ significantly (figure 4*a*).

**Figure 5.**
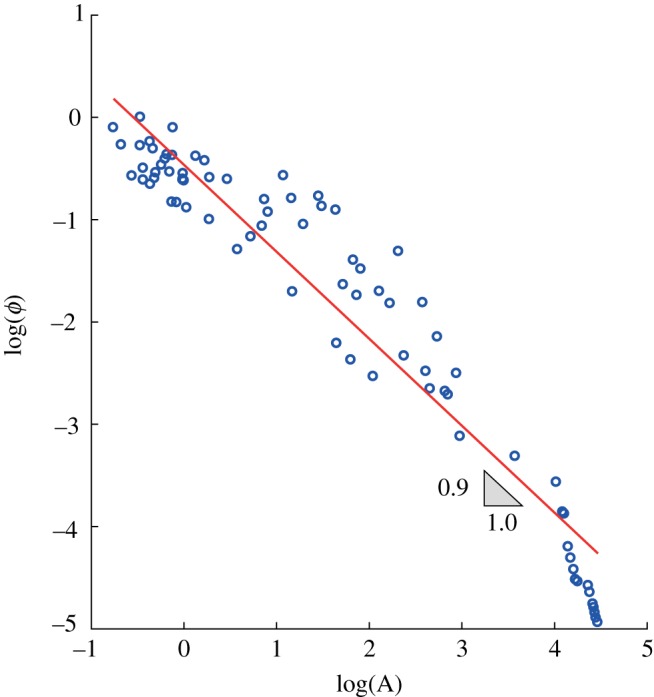
Scaling exponent of the runoff coefficient *ϕ* for 〈*P*〉 = 150 mm/season and λ_*P*_ = 1 d^−1^.

**Figure 6.**
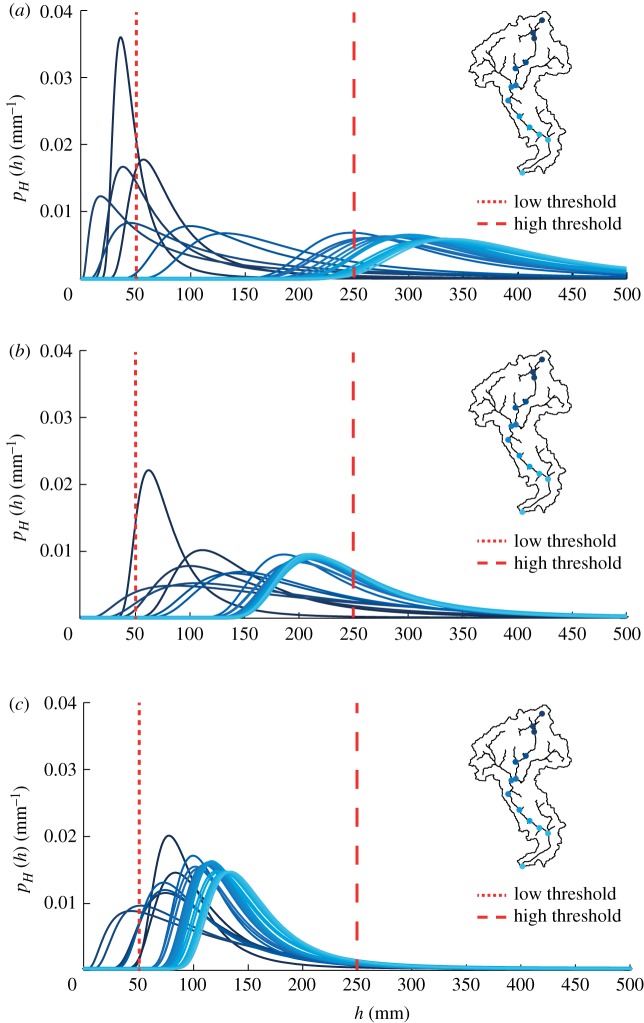
Patterns of stage PDFs along the network with 〈*P*〉 = 150 mm/season and (*a*) λ_*P*_ = 0.1 d^−1^, (*b*) λ_*P*_ = 0.5 d^−1^ and (*c*) λ_*P*_ = 1 d^−1^.

**Figure 7.**
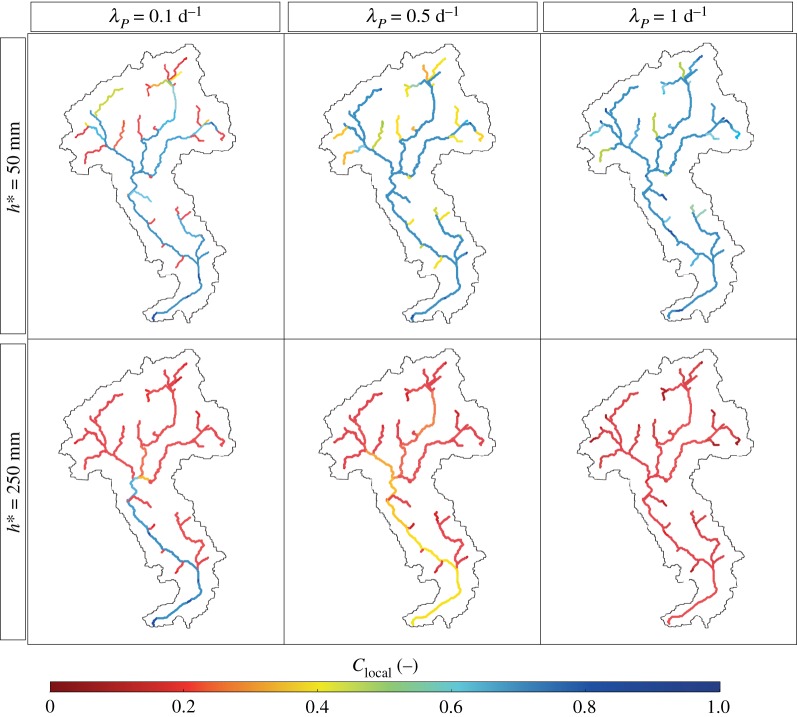
Spatial variability of local connectivity under a dry climate (〈*P*〉 = 150 mm/season and uniform 〈PET〉 = 3.5 mm d^−1^) for increasing rainfall frequencies λ_*P*_ and assuming different stage thresholds $h^{*}$.

The mean water depth, 〈*h*〉, generally increases with the drainage area (figure 4*c*), as a by-product of the Leopold and Maddock scaling relation (equation (2.7)) [57]. However, 〈*h*〉 slightly increases with A when λ_*P*_ = 1 d^−1^ as long as *ϕ* decreases with A as a power law with an exponent *β* close to 1 (figure 5). Therefore, when *β* approaches 1 the mean depth 〈*h*〉, which scales as [*A*^1−*β*^]^*δ*^, tends to remain constant throughout the network. When 〈*h*〉 remains nearly uniform along the network, the connectivity is affected by second-order moments of the stage PDF. In particular, for increasing λ_*P*_ the coefficient of variation of the stage distribution decreases, and the hydrological connectivity becomes a function of the relationship between 〈*h*〉 and $h^{*}$. Figure 6 shows the water stage PDFs for increasing frequencies of rainfall events, assuming 〈*P*〉 = 150 mm/season. For high rainfall frequency (figure 6*c*), the pronounced decrease of *ϕ* with A promotes high probabilities of relatively small water stages (〈*h*〉 ≃ 150 mm) in downstream sites. This strongly reduces the connectivity when $h^{*}>250$ mm. Therefore, in most circumstances, the runoff coefficient represents a key factor governing the spatial patterns of the probability distribution of the water stage along the network, and the ensuing connectivity. However, hydrological connectivity is also strongly dependent on the stage threshold $h^{*}$. Generally, low thresholds ($h^{*}=50$ mm) produce high connectivity everywhere along the network regardless of λ_*P*_. Higher thresholds ($h^{*}=250$ mm), instead, produce high connectivities in downstream sites (*C*_local_ = 0.7−1) only for low frequency of rainfall (λ_*P*_ = 0.1 d^−1^). Conversely, very low connectivities are observed throughout the network (*C*_local_ < 0.1) for higher values of λ_*P*_ (λ_*P*_ = 1 d^−1^). Therefore, under the same mean precipitation and for different values of λ_*P*_, different stage thresholds produce heterogeneous patterns of connectivity along the network (figure 7).

**Figure 8.**
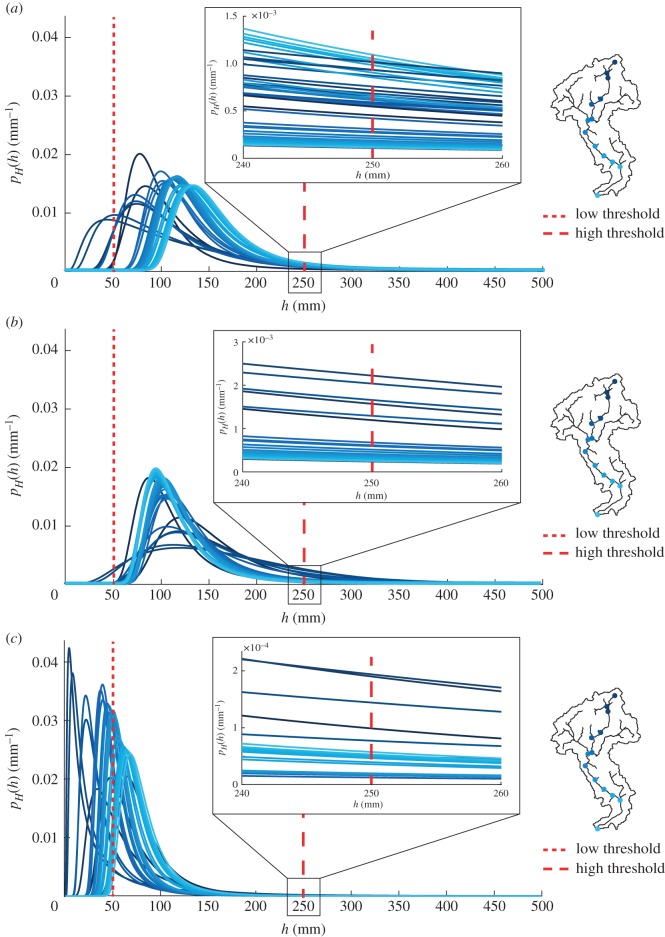
Patterns of stage PDFs along the network with 〈*P*〉 = 150 mm/season, λ_*P*_ = 1 d^−1^ and (*a*) uniform 〈PET〉, (*b*) 〈PET〉 increasing downstream and (*c*) 〈PET〉 decreasing downstream.

**Figure 9.**
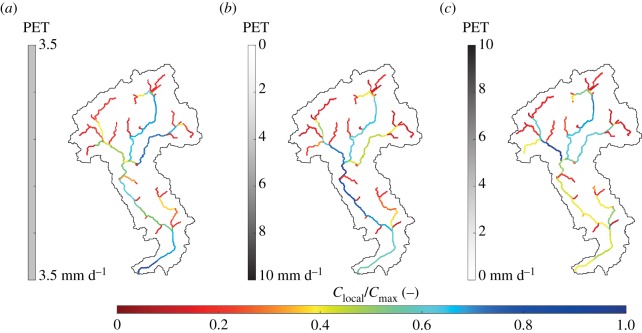
The ratio of the local connectivity to the maximum local connectivity assuming $h^{*}=250$ mm, 〈*P*〉 = 150 mm/season and (*a*) uniform 〈PET〉, (b) 〈PET〉 increasing downstream and (*c*) 〈PET〉 decreasing downstream.

Although the spatial variability of evapotranspiration does not affect the average connectivity of the network, the impact of PET patterns on stage PDFs and local connectivity in dry climatic conditions is noticeable. Figure 8 shows the probability distributions of water stages along the network for three different evapotranspiration patterns. When 〈PET〉 is assumed to be spatially uniform, 〈*h*〉 increases in downstream sites as driven by the increase in the drainage area. If 〈PET〉 is assumed to increase downstream, 〈*h*〉 slightly decreases along the network because the increase of spatially averaged PET from upstream to downstream sites enhances the decrease of the runoff coefficient for increasing contributing areas (figure 8*b*). The increase of 〈*h*〉 for larger contributing areas becomes less pronounced when 〈PET〉 is assumed to decrease downstream. Spatial patterns of 〈PET〉 affect the tail of *p*_*H*_(*h*), which is a second-order control on connectivity patterns. The probability of high water stages is reduced when 〈PET〉 is spatially variable (insets of figure 8*b*,*c*), with a reduction of connectivity especially in downstream sites. As a consequence, the same stage threshold used with different PET patterns produces different spatial distributions of hydrological connectivity at a local scale (figure 9). Overall, the analysis indicates the emergence of unexpected spatial patterns of connectivity induced by patterns of evapotranspiration, especially under arid climatic conditions. High values of local connectivity are observed not only in downstream sites (where the mean stage is typically higher) but also in river reaches located in the middle of the network (figure 9*b*,*c*).

### 3.3. Ecological value of hydrological connectivity

**Figure 10.**
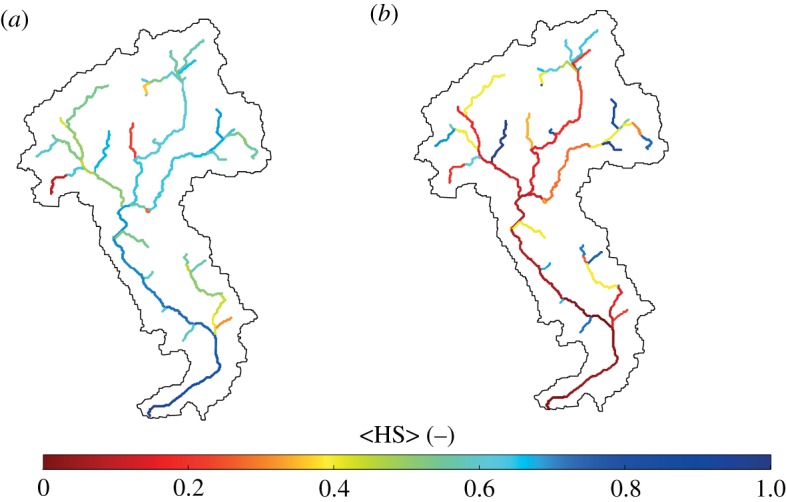
Spatial variability of habitat suitability under (*a*) a dry climatic scenario with 〈*P*〉 = 150 mm/season and (*b*) a wet climatic scenario with 〈*P*〉 = 350 mm/season.

In this section, we investigate how the habitat availability and the hydrological connectivity interact in response to space–time variability of climatic attributes. As a proof of concept, we refer here to the specific example of spawning sites for Atlantic salmon, and their connectivity to the catchment outlet. The spatial distribution of spawning sites for Atlantic salmon is modelled according to the assumptions discussed in appendix A. Under these assumptions, the mean habitat suitability 〈HS〉 is strongly dependent on the mean precipitation and streamflow. For very arid climates (〈*P*〉 = 150 mm/season) downstream reaches are more suitable for spawning, whereas under wetter climatic conditions the higher habitat suitability is located in the headwaters (figure 10).

To investigate the interaction between the spatial distribution of fish habitat suitability and hydrological connectivity as driven by flow regimes, we introduce the concept of outlet connectivity, which is a useful metric to evaluate the accessibility of spawning sites from the outlet in a river network. The outlet connectivity, *C*_out_, is calculated using equation (2.11) with specific reference to the outlet. *C*_out_ expresses the probability for the outlet to be connected with all network nodes, assuming that each node represents a patch of suitable habitat where individuals can reproduce and survive. Thus, direct connections between nodes are migration links between patches. However, in order to preserve the ecological function of rivers, the hydrological connectivity must be guaranteed, especially in those nodes whose ecological value is larger (in our example, the nodes where the habitat suitability is larger). Thus, the ecological connectivity of the outlet, *C*_eco_, can be calculated by weighting each outlet–node path using a weight proportional to the mean habitat suitability of the node,

3.1 Ceco=∑n=1n≠outNC(n→out) ⟨HS⟩n∑n=1n≠outN⟨HS⟩n.

Then, a suitability index SI is used to assess the impact of hydrological dynamics on ecological processes. SI is calculated as the ratio *C*_eco_/*C*_out_. If most ecologically suitable sites are located in nodes that are mostly connected with the outlet, SI > 1. On the other hand, SI < 1 when the most suitable sites are less hydrologically connected to the outlet. Key results of the application of the model under different climatic settings and connectivity thresholds are summarized in table 1.

**Table 1.** Outlet connectivity, effective connectivity and suitability index for dry and wet climatic settings as a function of stage water thresholds.

threshold (mm)	climate	*C*_out_ (−)	*C*_eco_ (−)	SI = *C*_eco_/*C*_out_ (−)
$h^{*}=50$	dry	0.80	0.80	1.00
	wet	0.85	0.70	0.80
$h^{*}=200$	dry	0.05	0.07	1.40
	wet	0.50	0.20	0.40

Dry: 〈*P*〉 = 150 mm/season; λ_*P*_ = 0.5 d^−1^. Wet: 〈*P*〉 = 350 mm/season; λ_*P*_ = 0.5 d^−1^.

Low stage thresholds produce high values of connectivity at local scale in both dry and wet climates, as the probability to observe water depths larger than 50 mm is relatively high everywhere in the network. Thus, the probability of the outlet being connected to the other nodes is high (*C*_out_ ≥ 0.80). In this case, most suitable sites (that are located downstream when the climate is dry or upstream when the climate is wet) are properly connected to the outlet, and SI values are close to 1 under both climatic scenarios. With larger stage thresholds ($h^{*}=200$ mm), the most connected stream reaches are generally located close to the outlet, where the mean water stage is higher. In this case, the outlet connectivity is severely reduced and *C*_out_ under the dry climate is 10 times smaller than the value obtained under the wet scenario. Although the outlet is insufficiently connected with the entire network, when precipitation is low (dry scenario), the most suitable sites are effectively connected to the outlet since they are located downstream (SI > 1). On the other hand, when precipitation is high, the most suitable reaches are located in the headwaters, which are poorly connected to the outlet. Thus, even though the overall values of *C*_eco_ and *C*_out_ are higher than those obtained in the dry scenario, SI < 1. This implies that, for relatively high thresholds, spawning sites are less accessible under wet climatic conditions. This simple example shows that, depending on the type of climate, the stage threshold and the spatial distribution of habitats, the emerging patterns of connectivity can either promote or limit ecological function of river networks.

## 4. Discussion

The ecological function of rivers is guaranteed by the physical connection between network nodes, which is driven by hydrological processes. Although connectivity metrics commonly found in the literature describe the spatial configuration of the network [39,41], a causal connection between river network connectivity and first-order climatic and hydrological drivers is missing. To fill this gap, the metrics proposed in this paper quantify the physical connection of the network on the basis of the hydrological state of the system. Our results indicate that the spatial variability of reach-scale connectivity might be controlled by the spatial and temporal distribution of climatic variables. Precipitation distribution, in terms of rainfall frequency and intensity, and spatial patterns of evapotranspiration concur to define the fraction of the hydrological network available for biological dispersion. Frequently, river networks in arid environments may be hydrologically disconnected because of insufficient water flows in relevant portions of the network. Moreover, spatial gradients of climatic properties influence the hydrological response and the connectivity of catchments whose size is larger than the integral scale of the relevant climatic heterogeneity. Therefore, spatial patterns of climate are likely to alter existing scaling properties of drainage networks inferred through purely geomorphological approaches [66].

**Figure 11.**
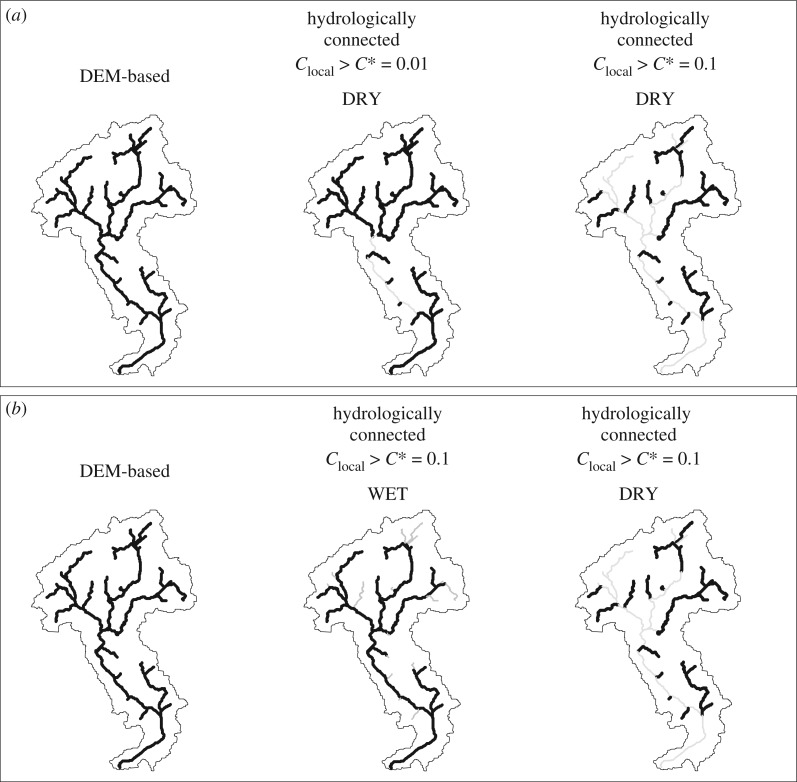
Comparison between a digital elevation model (DEM)-based network and hydrologically connected networks obtained using different connectivity thresholds C*; (*a*) C* = 0.01 and C* = 0.1 in dry climatic conditions and (*b*) C* = 0.1 in wet and dry climatic conditions.

Although the general influence of the hydrological connectivity on fauna migratory dynamics has been already documented in the literature [14,15,17], quantitative assessments of ecologically relevant stage thresholds remain problematic. In our framework, a critical connectivity threshold can be introduced to identify the likelihood of hydrological conditions favourable to migratory movements. In particular, we shall assume that two nodes with a local connectivity lower than a given threshold $C^{*}$ are physically disconnected because the likelihood of hydrological conditions favourable to species movement is too low. Lower values of $C^{*}$ are thus associated with greater efficiency during migration. Our simulations evidence that the shape of the connected network might be significantly altered by the underlying hydrological processes. This is represented in figure 11, which shows the shape of the connected network under different scenarios, whenever all the reaches with $C_{\mathrm{local}}<C^{*}$ are removed from the original network. During the dry season a low connectivity threshold ($C^{*}=10^{-2}$) breaks the network into two disconnected parts (figure 11*a*, centre). Larger portions of the main river channel are progressively excluded by increasing the connectivity threshold (figure 11*a*, right). The shape of the network is also modified when different climatic conditions are considered (figure 11*b*). Interestingly, under a wet climate the connectivity in the upper extent of the headwaters region is compromised; conversely, the main channel gradually disappears in the dry climate as the connectivity is too low. This dynamic behaviour of the flowing network might have a crucial impact on ecological models for species dispersion and propagation of waterborne diseases [3]. The proposed approach provides a quantitative framework that allows the description of the main hydrological causes and ecological consequences of hydrological dynamics experienced by river networks in response to climatic forcing. As such, the method could be integrated into network transport models currently used in spatial ecology, allowing for the use of time-variant and locally disconnected network domains, of the type shown in figure 11.

This study focuses on spatial patterns of hydrological connectivity along individual river networks, assuming that the number of network nodes is fixed and constant in time. An *a priori* definition of the relevant drainage density is thus required. On the other hand, the application of the stochastic approach to estimate the flow regime requires a minimum contributing area *A*_min_, of the order of some square kilometres. Therefore, the use of homogeneous criteria to identify the stream network in different river systems should allow a fair comparison of the connectivity metrics proposed in this paper across different catchments.

This work exploits a probabilistic framework for the characterization of the spatial variability of streamflow regimes and water stage dynamics driven by external climatic forcing. The method incorporates a number of hydrological models of proven robustness and wide applicability [2,43,44,51,54]. Nevertheless, our model relies on a number of simplifying assumptions. The hydrological model assumes a one-to-one relationship between catchment storage and discharge, which is here inferred solely from geomorphic data. Moreover, the developed framework does not take into account space–time variations in the relationship between river width and depth along the network. The constant nonlinear relation used to derive water stages from discharges is an assumption that could be relaxed only whenever *in situ* measurements of the geometry of cross sections along the river are available. River bed is also assumed impermeable and possible interactions between the stream and the surrounding environment are neglected. Nevertheless, the model is mathematically sound, has a reduced number of parameters with a direct physical meaning and is computationally inexpensive. Therefore, the approach represents an interesting prospect for eco-hydrological spatially explicit studies.

## 5. Conclusion

In this paper, we propose an analytical approach where hydrological connectivity is explicitly linked to driving hydroclimatic variables and catchment properties through the emergent spatial patterns of streamflow regimes along river networks. The method is based on a stochastic generation of rainfall able to reproduce different climatic scenarios in terms of rainfall frequency, intensity and amount. Results confirm that precipitation regimes significantly impact the connectivity of river networks. Network connectivity typically increases by increasing the mean precipitation and the frequency of rainfall events. Under arid climatic conditions, network connectivity is higher for rare but intense events, of the type found in semi-arid regions.

Evapotranspiration is a key factor controlling the rate of decrease of the runoff coefficient along river networks, with noticeable effects on mean water stages and hydrological connectivity. A smooth decrease of the rainfall runoff coefficient generates increasing mean stages for larger drainage areas; vice versa, when the reduction in the runoff coefficient is faster (e.g. when rainfall events are frequent and when evapotranspiration is spatially variable, especially under arid conditions), the mean stage increases much slower downstream, making the connectivity dependent on the interplay between flow variability and the stage threshold $h^{*}$.

Our simulations show that spatial patterns of evapotranspiration strongly influence the variability of the hydrological connectivity along the network, without impacting the mean network connectivity.

The proposed framework helps to identify the physical controls on hydrological connectivity and their effect on ecological processes along river networks, as documented by the proof of concept pertaining to salmon migration discussed in §3.3. The analysis shows that, depending on the climate and the spatial variability of habitat suitability, the resulting connectivity patterns can either promote or limit the ecological function of river networks.

To provide a quantitative assessment of the impact of hydrological processes on the shape and the extent of ecologically connected reaches, we have analysed the changes in the topological configuration of the river network, when all the streams with insufficient connectivity are removed. Our analysis reveals that under arid climates the main channel may become disconnected from the tributaries, whereas under wet climates river networks tend to shrink from the headwaters. Therefore, the shape of connected networks can be significantly impacted by the underlying hydrological dynamics.

The general mathematical formulation proposed in this paper encourages the application to other synthetic networks and to real-world case studies. The method offers a robust basis to assess the ecological impacts of streamflow variability in rivers, and it is thus suited to be coupled with spatially explicit ecological network models.

## Data accessibility

Data are available from the Dryad Digital Repository: http://dx.doi.org/10.5061/dryad.4r695ph [70].

## Appendix A

This section describes the estimation of the flow model parameters and the simulation set-up to predict spatial patterns of hydrological connectivity across catchment scales in a theoretical case study. At first, rain cells are generated using the three-dimensional Poisson rainfall model at the catchment scale. Table 2 shows the parameter ranges used to reproduce different climatic scenarios. Parameters of the spatially distributed rainfall model are assumed by referring to the literature values in terms of rainfall frequency and rainfall intensity. Moreover, the integral scale of the climatic heterogeneity is assumed to be smaller than the catchment size as the cells’ radii are meant to represent convective rainfall. Note that, in the proposed framework, a periodic space domain is used to reduce bias from edge effects.

**Table 2.** Model parameters for the rainfall generation model.

parameter	symbol	value	units
temporal density	λ_*t*_	1.5–11	(d^−1^)
spatial density along the *x* direction	λ_*x*_	0.7–1.2	(m^−1^)
spatial density along the *y* direction	λ_*y*_	0.7–1.2	(m^−1^)
mean rain cell depth	〈*ζ*〉	15–4500	(mm)
mean rain cell radius	〈*r*〉	1–2	(km)

The average frequency of rainfall events along the network, λ_*P*_, is estimated by evaluating the relative fraction of rainy days (rain depth greater than 1 mm) during the considered time period in the upstream contributing area of each node. The mean rainfall depth, *α*, is then calculated for each realization of the rainfall model as the spatially averaged rain intensity during wet days (i.e. the volume of rainfall is divided by the number of wet days and the catchment area drained by each node). The frequency of the effective rainfall, λ, is then estimated considering the censoring effect of the soil catchment that results in the reduction of flow-producing rate controlled by evapotranspiration. To this aim, spatially distributed values of PET (i.e. PET(X)) are used. The runoff coefficient *ϕ* is estimated using equation (2.3), considering the potential evapotranspiration as a spatially averaged value in the upstream contributing catchment. λ is then calculated as the product *ϕ*λ_*P*_ for every node of the network. The literature values of porosity *n*, rooting depth *Z*_*r*_, soil moisture at saturation *s*_1_ and wilting point *s*_*w*_ are considered and incorporated in equation (2.3) (table 3).

**Table 3.** Water balance parameters.

parameter	symbol	value	units
soil moisture at saturation	*s*_1_	0.5	(−)
wilting point	*s*_*w*_	0.2	(−)
porosity	*n*	0.3	(−)
rooting depth	*Z*_*r*_	130	(mm)

Parameters defining the storage–discharge relations are then derived by river network analysis as detailed in §2.1.3. The river network estimation is derived from a representative digital elevation model from which eight-flow direction and flow accumulation maps are calculated to identify stream channels; then, the flow accumulation threshold equal to 25 unit area is applied to extract the channel network. As specific morphological requirements are needed to estimate the recession parameter *a* along the network (e.g. at least three junctions are required upstream to each considered node), a finer resolution ancillary network with a larger drainage density is used to calculate the recession exponent in the headwaters. Subsequently, considering both climatic and geomorphic features of the catchment, the recession constant *K* is calculated as *K* = *θ*(*α*λ)^1−*a*^ using a constant value of *θ* equal to 0.2 d^−1^ [51].

The streamflow distribution calculated pointwise using equation (2.6) is finally used as the input to derive the spatial and temporal variability of water depth by applying equation (2.7). The parameters of the nonlinear discharge–depth relation are assumed to be constant along the river network. Connectivity metrics are then calculated using two sets of critical stages: (i) low stage threshold (i.e. $h^{*}=5$ cm), which is assumed to be associated with smaller general species, such as bacteria or propagules, requiring small amounts of water to disperse; (ii) high stage threshold (i.e. $h^{*}=25$ cm), which is representative of fish species that need relatively high water stages for migration [67]. A list of threshold examples associated with adult salmon and trout taken from the literature is reported in table 4.

**Table 4.** Example illustrating the minimum depth requirements for successful upstream migration of adult salmon and trout.

species of fish	minimum depth (m)
pink salmon	0.18
chum salmon	0.18
coho salmon	0.18
sockeye salmon	0.18
spring chinook salmon	0.24
summer chinook salmon	0.24
fall chinook salmon	0.24
steelhead trout	0.18

Data from [68].

In order to evaluate the ecological function of the hydrological connectivity the habitat distribution is also included in the simulation via equation (2.13), with specific reference to salmon migration towards the headwaters. Several studies pertaining to Atlantic salmon, based on experimental data and hydraulic models [64,69], have proposed a range of variability for parameters A, B and C in equation (2.13). The minimum and the maximum value for each parameter are reported in table 5. In view of the uncertainty associated with the parameters controlling habitat suitability distribution, which depends on bed morphology, water quantity, water quality and size species, we considered intermediate values of the literature ranges. Parameters are assumed constant throughout the river network. Different parameter combinations were also tested without significant changes in the results presented in this paper.

**Table 5.** Minimum and maximum values of the habitat distribution model. The parameter set used in the simulation is also reported in the table (penultimate column).

parameter	min.	max.	assumed	units
A	0.9	1.1	1.0	(−)
B	2.5	12.0	5.6	(L T^−1^)
C	0.5	1.3	1.3	(T L^−1^)
